# Does Educational Assortative Mating Matter? Parental Education Matching and Children’s Academic Achievement in Urban China

**DOI:** 10.3390/bs16071235

**Published:** 2026-07-20

**Authors:** Jiazhe Li, Xuechun Wang, Jijun Yao, Shike Zhou

**Affiliations:** 1 China Rural Education Development Institute, Faculty of Education, Northeast Normal University, Changchun 130024, China; lijiazhe116@nenu.edu.cn; 2 Department of Educational Administration and Policy, Faculty of Education, The Chinese University of Hong Kong, Hong Kong 999077, China; 3 School of Educational Science, Nanjing Normal University, Nanjing 210097, China; yaojijun_njnu@163.com; 4 Jiangsu Basic Education Quality Monitoring Center, Nanjing 210013, China

**Keywords:** parental education, academic performance, urban primary school students, hierarchical linear model, path analysis

## Abstract

Parental education, as institutionalized family cultural capital, is significantly associated with children’s academic performance. Using provincial-level academic quality monitoring data, this study employs hierarchical linear models and path analysis to examine how parental education levels and matching patterns relate to urban primary school students’ academic performance. Key findings: Higher overall parental or paternal education is positively associated with performance; however, maternal education exhibits an inverted U-shaped relationship. Compared to both parents non-highly-educated families, students perform significantly better when both parents are highly educated or when fathers are highly educated and mothers are not, but significantly worse in families where the mother is highly educated while the father is not. Path analysis indicates that the negative association in mother-hypergamy families is partly statistically associated with reduced paternal learning involvement, which in turn is related to heightened academic pressure and poorer outcomes. The study suggests that the lower academic performance observed in mother-hypergamy families may be partly associated with reduced paternal learning involvement, highlighting the importance of family educational configuration and balanced co-parenting dynamics. These findings suggest that the association between parental education and children’s academic performance is not straightforward. While consistent with cultural reproduction and family investment perspectives, the detrimental effect of the mother-hypergamy pattern highlights the importance of family configuration and points to the potential roles of role conflict, co-parenting dynamics, and expectation-pressure mechanisms that warrant direct empirical investigation in future research.

## 1. Introduction

In recent years, declining educational returns among China’s new middle class have sparked widespread discussion. The “2021 New Middle Class White Paper” reveals that children of graduate and highly educated parents generally earn more than children of parents with lower education. Cultural reproduction theory (Bourdieu, 1986) and human capital transmission frameworks (Becker & Tomes, 1986) suggest that highly educated parents possess advantageous cultural and economic capital that should promote children’s academic success through active educational participation. However, in China’s current educational reality, many highly educated parents remain anxious about their children’s academic performance. Why do children of highly educated parents show a lack of interest in learning or poor academic performance?

Most existing research incorporates parental education into the family socioeconomic status or analyzes the impact of parents’ average education on children’s cognitive abilities, mental health, and social behavior (Minello & Blossfeld, 2017; A. L. Park et al., 2013). Some studies emphasize the impact of maternal education on children’s development (Cuartas, 2022), but few studies have carefully analyzed whether children of highly educated parents indeed perform better academically. Additionally, the consequences of educational assortative mating extend beyond the couple to shape broader family outcomes. Qing (2025), using data from the China Family Panel Studies (CFPS), demonstrated that spousal educational matching patterns significantly influence fertility behavior, with highly educated homogamous couples exhibiting the lowest fertility levels. Becker’s quantity-quality tradeoff model posits that, given finite resources, parents allocate them between child quantity and quality. Highly educated parents, as beneficiaries of human capital investment, tend to focus resources on a smaller number of offspring to achieve superior educational outcomes. Furthermore, within educational homogamy, higher spousal education is associated with higher parental expectations for children’s education, suggesting that such couples both bear fewer children and attach greater importance to their children’s academic success.

Different parental educational matching patterns may result in variations in children’s academic performance. According to assortative mating theory (Schwartz, 2013; Mare, 2000), marriage patterns based on educational attainment have significant implications for family resource allocation and child development. Whether in China or Western countries, traditional marriage matching patterns in terms of socioeconomic status primarily manifest as two models: “male hypergamy” and “homogamy” (Ma et al., 2019). However, with the reversal of gender differences in education, the “female hypogamy” marriage matching pattern has been challenged; China has witnessed the emergence of the “male hypogamy” model, with its proportion gradually matching that of the traditional “male hypergamy” model (Y. Hu, 2016).

Currently, little research has explored how different parental educational matching patterns affect children’s academic performance. This study classifies parental educational matching patterns into four types: both parents highly educated, father highly educated and mother non-highly educated, mother-hypergamy, and both parents non-highly educated, to discuss this issue. It is expected that this study can provide a useful reference for family education practices with different parental educational matching patterns.

### 1.1. Theoretical Foundation

This study draws on three complementary theoretical frameworks. First, Bourdieu’s (1986) cultural reproduction theory posits that parental education, as institutionalized family cultural capital, transmits advantages to children through embodied dispositions, resource provision, and educational expectations. In the Chinese context, highly educated parents often adopt “educational downward mobility avoidance” strategies to maintain class status across generations (Hou, 2015). This framework provides the overarching rationale for expecting a positive association between parental education and children’s academic performance.

Second, the family investment model (Conger et al., 2010; Yeung et al., 2002) articulates specific mechanisms through which socioeconomic resources translate into child outcomes via material and process investments—particularly parental time, interaction quality, and learning involvement (Sohr-Preston et al., 2013). Highly educated parents tend to acquire more child development knowledge and apply more effective parenting strategies (Conger & Donnellan, 2007). This model directly informs our examination of paternal and maternal learning participation as potential mediating pathways.

Third, the family stress model (Conger et al., 2010; Gard et al., 2020) provides a counterbalancing perspective. Excessive parental expectations and intrusive involvement can generate stress and anxiety that undermine children’s performance (Finkelstein et al., 2007; Kocsis et al., 2025). This lens helps explain why children of highly educated mothers, particularly in families where fathers are less educated, may experience heightened academic pressure and poorer outcomes, despite the apparent advantages of maternal education.

Together, these frameworks suggest that the effects of parental education on children’s academic outcomes are not simply additive but depend critically on how parents’ educational resources are configured within the family and on the quality and intensity of parental involvement. We therefore posit that while higher overall parental education is expected to be positively associated with performance, the specific matching patterns may yield more complex results, particularly when maternal education exceeds paternal education.

### 1.2. The Relationship Between Parental Education and Children’s Academic Performance

Most domestic and international research shows that parental education is significantly positively correlated with children’s academic performance (Dewey et al., 2000; De Haan & Plug, 2011; H. Hu et al., 2012; Lin, 2015; Sun & Yan, 2015). For example, using TIMSS data, Wößmann (2005) found that after controlling for relevant covariates, parental education has a significant positive impact on the academic performance of students in Hong Kong (China), South Korea, Japan, Singapore, and Thailand. Ludeke et al.’s (2021) analysis of parental education and children’s educational attainment in Danish twin and adoptive families found that highly educated parents can enhance their children’s academic performance through both genetic and non-genetic pathways. Highly educated parents possess stronger human capital investment capabilities and efficiency; they have the ability to provide their children with more comfortable living conditions and learning environments, and by creating a good family atmosphere and participating in their children’s learning and life with high quality, they promote their children’s achievement of good academic performance (Plug & Vijverberg, 2005; Guryan et al., 2008). Yao and Chen’s (2020) research also shows that highly educated mothers have a positive impact on their children’s years of education through income effects, quantity-quality substitution effects, and auxiliary effects.

However, some individual studies have found that after controlling for relevant variables, maternal education has no significant impact on children’s academic performance. For instance, A. Park and Hannum (2001), based on data from a rural basic education survey in Gansu, China, found that after controlling for teacher characteristics and class size, father’s education has a significant positive impact on children’s mathematics scores but no significant impact on Chinese scores, while mother’s education has no significant impact on either children’s Chinese or mathematics scores. Z. Li’s (2013) research also found that father’s education has a significant impact on children’s Chinese, mathematics, and English scores, while mother’s education has no significant impact on any of these subjects. Some individual studies have even found that maternal education is significantly negatively correlated with children’s academic performance. For example, Y. Hu’s (2007) research found that after controlling for variables such as class size, teacher education, and teaching experience, maternal education is significantly negatively correlated with both mathematics and Chinese scores of sixth-grade primary school students. The author suggests that this may be related to the generally low education levels of mothers in rural areas of western China, making it difficult to provide substantive guidance for their children’s learning. Moreover, the higher the mother’s education, the greater the probability of working away from home, making it difficult to regularly pay attention to and participate in children’s learning. Additionally, the author emphasizes that there is collinearity between fathers’ and mothers’ education, making it inappropriate to include both in the regression model simultaneously. Based on this, most research generally confirms that overall parental education level or father’s education level is significantly positively correlated with children’s academic performance, but regarding the relationship between maternal education and children’s academic performance, existing research has not reached a consistent conclusion. This study proposes to further examine the linear and nonlinear relationships between overall parental education, father’s education, or mother’s education levels and children’s academic performance. Accordingly, the following research hypotheses are proposed for testing:

**H1:**  *After controlling for relevant covariates, parental education shows a significant positive correlation with urban primary school students’ academic performance*.

**H1a:**  *After controlling for relevant covariates, the overall level of parental education shows a significant positive correlation with urban primary school students’ academic performance*.

**H1b:**  *After controlling for relevant covariates, father’s education level shows a significant positive correlation with urban primary school students’ academic performance*.

**H1c:**  *After controlling for relevant covariates, mother’s education level shows a significant positive correlation with urban primary school students’ academic performance*.

Furthermore, some research has found that children of highly educated parents actually experience lower educational attainment or poor academic performance. This may be because children of highly educated parents indulge in enjoyment and fail to properly utilize the conditions created by their parents (Spagat, 2006). Although highly educated parents typically have higher-quality educational participation, their work may be busier, leaving them no time to care for, accompany, and educate their children. While some studies attribute this to children’s indulgence or parents’ time constraints, Kocsis et al. (2025) offer a complementary explanation through the lens of helicopter parenting. Their systematic review reveals that excessive parental control, even when well-intentioned, can reduce student autonomy, undermine self-regulated learning, and increase school burnout. This suggests that the quality and intensity of parental involvement, rather than its mere presence, critically moderate the relationship between parental education and children’s academic outcomes.

In addition to discussing the impact of a father’s or mother’s education level on children’s academic performance, some research has also discussed the impact of parental educational gaps (father’s education higher than mother’s, father’s education lower than mother’s, and parents with equal education) on children’s educational levels, finding that compared to other families, “homogamous” parental education is more conducive to the intergenerational transmission of human capital (Zhou et al., 2018). Based on this, this study proposes to classify fathers’ or mothers’ education as high education or non-high education and examine the impact of different parental educational matching patterns on children’s academic performance. Therefore, we propose the following research hypotheses for testing:

**H2:**  *Controlling for relevant variables, there are significant differences in the academic performance of urban primary school students with different parental educational matching patterns*.

**H2a:**  *Compared to both parents having non-higher education, urban primary school students whose parents both have higher education have significantly better academic performance*.

**H2b:**  *Compared to both parents having non-high education, urban primary school students whose father has high education and whose mother has non-high education have significantly better academic performance*.

**H2c:**  *Compared to both parents having non-high education, urban primary school students whose father has non-high education and whose mother has high education have significantly better academic performance*.

## 2. Materials and Methods

### 2.1. Data Source

The data for this study were derived from the 2020 Provincial Basic Education Student Academic Quality Monitoring Project, a large-scale biennial survey administered by the Jiangsu Basic Education Quality Monitoring Center in China. The project employed a two-stage stratified sampling design to survey school principals, teachers, parents, and students. In the first stage, schools were stratified by urban/rural location and school type, and a random sample of schools was selected proportionally. In the second stage, within each selected school, one or more classes were randomly chosen, and all students in the participating classes were invited to complete the student questionnaire. In 2020, a total of 214,205 fifth-grade students participated in the survey. This study focuses on urban primary school student samples. Through data organization, matching, and elimination of samples that failed attention check items, had missing important information, or contained obvious data anomalies, the study ultimately obtained 104,975 valid samples. Among them, 48% were female, 43% were only children, and 2% were boarding students. The data used in this study were provided to the research team as strictly anonymized secondary data under a formal institutional data-use agreement with the Jiangsu Basic Education Quality Monitoring Center; no personally identifiable information was accessible to the authors.

### 2.2. Model Specification

The data in this study are cross-sectional data with multiple levels including students and schools. Based on the nested characteristics of the data, we employ both ordinary least squares (OLS) regression and hierarchical linear models for estimation. The data analysis is conducted through Stata/MP 18.0. Referring to existing research (Y. Hu & Yuan, 2021; Jhang & Lee, 2018; Ndlovu, 2018; Xue et al., 2020), this study includes as control variables at the student individual level: gender, whether the student is an only child, and whether the student is a boarding student; and at the school level: students’ average family economic status, teachers’ average teaching experience and its squared term, and school support level (specific variable descriptions are shown in Table 1). The specified models are as follows:

#### 2.2.1. Ordinary Least Squares Regression Model

The ordinary least squares multiple regression model takes the following form:

$${SCO}_{i}=b_{0}+b_{1}\cdot {PED}_{i}+b_{2}\cdot {GEN}_{i}+b_{3}\cdot {SIN}_{i}+b_{4}\cdot {LSC}_{i}{\;+\;b}_{5}\cdot {SESM}_{i}+{\;b}_{6}\cdot {TEAM}_{i}\;+{\;b}_{7}\cdot {{TEAM}_{i}}^{2}+{\;b}_{8}\cdot {SSL}_{i}+\mu_{i}$$

where ${SCO}_{i}$ represents the academic performance of student i, ${PED}_{i}$ represents parental education level or parental educational matching patterns, ${GEN}_{i}$ indicates student gender, ${SIN}_{i}$ indicates whether the student is an only child, ${LSC}_{i}$ indicates whether the student is a boarding student, ${SESM}_{i}$ represents the average family socioeconomic status at the school level, ${TEAM}_{i}$ represents the average teacher’s teaching experience and ${{TEAM}_{i}}^{2}$ its squared term, ${SSL}_{i}$ represents the school support level, $b_{0}$ is the intercept term, $b_{1}$ through $b_{8}$ are the regression coefficients for each variable, and $\mu_{i}$ is the random error term.

This model serves to estimate the overall effects of parental education variables on urban primary school students’ academic performance while controlling for other relevant factors. By including control variables at both the student and school levels, the OLS regression model helps to reduce omitted variable bias and more accurately identify the net impact of parental education. The model allows us to examine whether higher levels of parental education or specific parental educational matching patterns are significantly associated with better student academic outcomes after accounting for confounding factors such as student gender, only child status, boarding status, family economic conditions, teacher experience, and school support levels. The coefficient $b_{1}$ is of primary interest as it captures the relationship between parental education and student academic performance. Additionally, the OLS regression results provide a baseline comparison for the hierarchical linear model estimates, allowing us to assess the robustness of our findings across different analytical approaches. However, it should be noted that the OLS regression assumes independence among all observations, which may not hold true when students are nested within schools, potentially leading to biased standard error estimates and incorrect statistical inferences.

#### 2.2.2. Hierarchical Linear Model

Given the nested structure of our data, where students are clustered within schools, we employ hierarchical linear modeling as our primary analytical strategy. The hierarchical linear model is specified as follows:

Level 1 (Student Level):

$${SCO}_{ij}=b_{0j}+b_{1j}\cdot {PED}_{ij}+b_{2j}\cdot {GEN}_{ij}+b_{3j}\cdot {SIN}_{ij}+b_{4j}{\cdot {LSC}_{ij}+\varepsilon }_{ij}$$

Level 2 (School Level):

$$b_{0j}=\gamma_{00}+\gamma_{01}\cdot {SESM}_{j}+\gamma_{02}\cdot {TEAM}_{j}+\gamma_{03}\cdot {{TEAM}_{j}}^{2}+\gamma_{04}\cdot {SSL}_{j}+\mu_{j}$$

where ${SCO}_{ij}$ represents the academic performance of student i in school j, ${PED}_{ij}$ represents the parental education level or educational matching pattern for student i in school j, ${GEN}_{ij}$ indicates gender, ${SIN}_{ij}$ indicates only child status, and ${LSC}_{ij}$ indicates boarding status. At the school level, ${SESM}_{j}$ represents the average family socioeconomic status of students in school j, ${TEAM}_{j}$ represents the average teaching experience of teachers in school j, ${{TEAM}_{j}}^{2}$ is its squared term, and ${SSL}_{j}$ represents the support level of school j. The parameter $b_{0j}$ represents the intercept for school j, indicating the average academic performance level of students in that school, while $b_{1j}$ through $b_{4j}$ represent the effects of student-level variables within school j. At the school level, $\gamma_{00}$ represents the overall average academic performance across all schools, $\gamma_{01}$ through $\gamma_{04}$ represent the effects of school-level variables on school average performance. The terms $\varepsilon_{ij}$ and $\mu_{j}$ represent random errors at the student and school levels respectively.

The hierarchical linear model addresses several critical analytical challenges in this study. Most importantly, it appropriately handles the nested data structure where students are clustered within schools, allowing us to decompose variance and separate between-student and between-school differences. This approach avoids statistical inference errors that could arise from ignoring the hierarchical nature of the data, such as underestimated standard errors and inflated Type I error rates. The model simultaneously estimates effects at multiple levels, enabling us to examine both individual student characteristics such as parental education and contextual school factors such as teacher experience and school support. Through the null model estimation, we can calculate the intraclass correlation coefficient to determine what proportion of variance in student academic performance is attributable to school-level differences. Our null model results indicate that school-level variance accounts for 22.7% of total variance in academic performance, with statistical significance (*p* < 0.001), demonstrating the necessity of employing hierarchical linear modeling rather than conventional regression approaches. By accounting for school-level random effects through the term $\mu_{j}$, the hierarchical linear model provides more accurate parameter estimates and standard errors compared to OLS regression, yielding more reliable statistical inferences for testing our research hypotheses regarding the impact of parental education levels and matching patterns on urban primary school students’ academic performance. The decomposition of effects across levels allows us to understand not only whether parental education matters, but also how school contextual factors may moderate or influence these relationships.

## 3. Results

### 3.1. Differences in Academic Performance of Urban Primary School Students with Different Parental Education Levels and Matching Patterns

**Figure 1:**
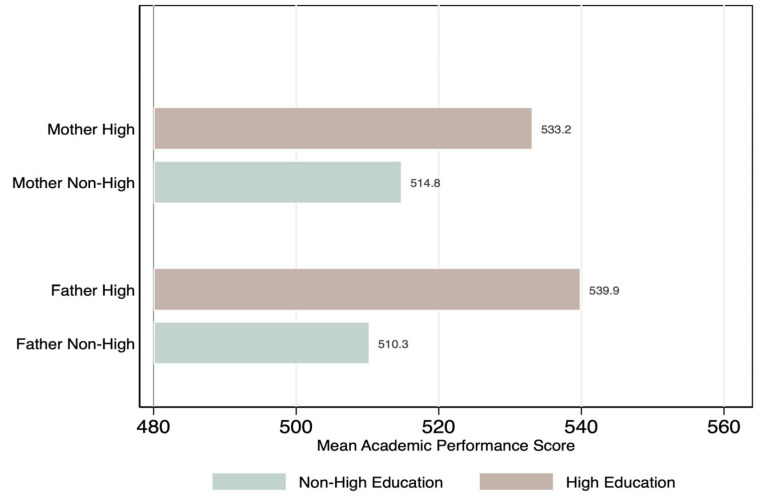
Differences in primary school students’ academic performance by parental education level.

Independent-samples *t*-tests were conducted to compare the academic performance of urban primary school students whose fathers or mothers had higher education versus those whose parents did not. As shown in Figure 1, children of highly educated mothers scored significantly (*p* < 0.001, Cohen’s d = 0.240) higher (M = 533.2) than children of non-highly educated mothers (M = 514.8). Similarly, children of highly educated fathers performed significantly (*p* < 0.001, Cohen’s d = 0.473) better (M = 539.9) than children of non-highly educated fathers (M = 510.3). The mean score gap between children of highly educated versus non-highly educated fathers corresponds to about 0.473 standard deviations, a difference that is roughly equivalent to the gap between adjacent quartiles of the achievement distribution. Similar results were obtained when the criterion for high parental education was redefined as graduate-level education or above (see Supplementary Materials for details).

**Figure 2:**
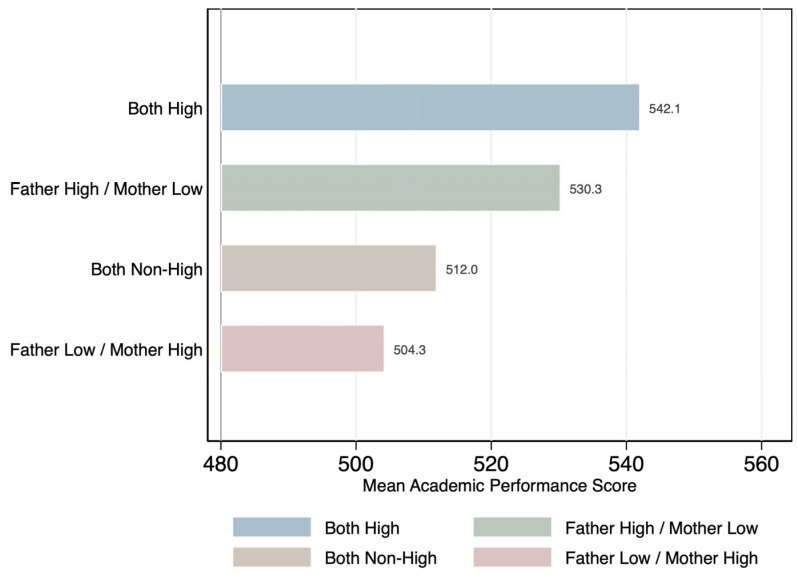
Differences in primary school students’ academic performance by parental educational matching patterns.

As shown in Figure 2, urban primary school students whose parents are both highly educated perform best academically, with mean scores of 542.1, followed by students whose father is highly educated and whose mother is non-highly educated (M = 530.3). However, the academic performance of urban primary school students whose father is non-highly educated and whose mother is highly educated is relatively poorer (M = 504.3), scoring even lower than students whose parents are both non-highly educated (M = 512.0).

We used one-way ANOVA and post hoc comparison methods to compare the academic performance of urban primary school students with different parental educational matching patterns. Results show that there are significant differences in the academic performance of urban primary school students with different parental educational matching patterns (F = 3476.88, *p* < 0.001), but they did not pass the homogeneity of variance test (*p* < 0.001), so Tamhane’s T2 method was selected for post hoc comparison. Results show that using both parents as non-highly educated as a reference, urban primary school students whose parents are both highly educated (Cohen’s d = 0.611) or whose father is highly educated and mother is non-highly educated (Cohen’s d = 0.359) have significantly better academic performance (*p* < 0.001), while urban primary school students whose father is non-highly educated and mother is highly educated (Cohen’s d = 0.148) have significantly worse academic performance (*p* < 0.001). Further pairwise comparisons using Tamhane’s T2 revealed that children with both highly educated parents performed significantly better than those with non-highly educated fathers and highly educated mothers (Cohen’s d = 0.774, *p* < 0.001). Likewise, children with highly educated fathers and non-highly educated mothers scored significantly higher than those with non-highly educated fathers and highly educated mothers (Cohen’s d = 0.486, *p* < 0.001). Moreover, children with both highly educated parents significantly outperformed those with highly educated fathers but non-highly educated mothers (Cohen’s d = 0.249, *p* < 0.001). Robustness checks using the graduate definition yield consistent results, reported in the Supplementary Materials.

To interpret these effect sizes in more practical terms, the 30-point advantage of the “both parents highly educated” group over the “both non-highly educated” group (d = 0.611) represents a meaningful gap in the achievement distribution. Even the relatively smaller effect for the “father non-highly educated/mother highly educated” group versus the reference group (d = 0.148) corresponds to a consistent and replicable pattern. However, given the large sample size, even small differences can achieve statistical significance. Therefore, these effect sizes should be interpreted with appropriate caution, and the above analysis did not control for relevant variables; subsequent regression analysis is still needed to further clarify the relationship between different parental education levels or different educational matching patterns and urban primary school students’ academic performance.

### 3.2. Estimation of the Impact of Parental Education and Its Matching Patterns on Urban Primary School Students’ Academic Performance

Null model results show school-level variation is statistically significant (*p* < 0.001), with ICC indicating 22.7% of total variance is attributable to school differences, justifying the use of hierarchical linear models (Raudenbush & Bryk, 2002; Xue et al., 2020).

Table 2 uses hierarchical linear models to estimate the linear and nonlinear impacts of different parental education levels on urban primary school students’ academic performance. The corresponding OLS regression estimation results are consistent with the HLM results. Model (1) shows that, controlling for relevant variables, father’s education level is significantly positively associated with urban primary school students’ academic performance (*p* < 0.001). Model (2) shows that after adding the quadratic term of father’s education, father’s education level and children’s academic performance show a nonlinear relationship, but within the given range of values (9–19), the higher the father’s education level, the better the children’s academic performance. This finding is consistent with H1b, aligning with cultural reproduction theory and the family investment model’s predictions that higher parental education enhances outcomes through cultural capital transmission and increased investments (Bourdieu, 1986; Yeung et al., 2002).

Model (3) shows that after controlling for relevant covariates, mother’s education level is significantly positively associated with urban primary school students’ academic performance (*p* < 0.001). Model (4) shows that after adding the quadratic term of mother’s education, urban primary school students’ academic performance shows a “first rising then declining” trend with increasing mother’s education level, i.e., there is an “inverted U-shaped” relationship between mother’s education level and children’s academic performance, and urban primary school students whose mothers have undergraduate education perform better, even better than those whose mothers have graduate education. This rejects H1c and suggests complexity beyond linear models. This finding may reflect the family stress model’s prediction that excessive educational expectations by highly educated mothers generate counterproductive pressures (Conger et al., 2010; Gard et al., 2020). As maternal education increases, labor market participation rises and marginal educational contributions decrease (Zhou et al., 2018), possibly explaining diminishing or negative returns at the highest education levels.

Model (5) shows that after controlling for relevant covariates, overall parental education level shows a significant positive relationship with urban primary school students’ academic performance (*p* < 0.01). Model (6) shows that after adding the quadratic term of overall parental education, urban primary school students’ academic performance shows a nonlinear relationship with overall parental education level, but within the given range of values (19–38), the higher the overall parental education level, the better the children’s academic performance. This verifies hypothesis H1a.

Table 3 uses OLS regression and hierarchical linear models to estimate the relationship between different parental educational matching patterns and urban primary school students’ academic performance. The estimation results are consistent across models: after controlling for relevant covariates, using both parents as non-highly educated as a reference, urban primary school students whose parents are both highly educated or whose father is highly educated and mother is non-highly educated have significantly better academic performance (*p* < 0.001), while urban primary school students whose father is non-highly educated and mother is highly educated have significantly worse academic performance (*p* < 0.001). This provides support for hypotheses H2a and H2b but rejects hypothesis H2c. We additionally performed robustness checks by redefining the criterion for high parental education as graduate-level education or above. The results from these alternative specifications remained essentially unchanged and consistent with those reported in Table 3, confirming the stability of our main findings (see Supplementary Materials).

Although the student-level pseudo-R^2^ values reported in Table 2 and Table 3 range from 0.139 to 0.162, indicating that parental education and its matching patterns explain a modest share of the total variance in academic performance, the regression coefficients nonetheless translate into tangible score differences. For instance, the coefficient for “both parents highly educated” (approximately 8–15 points depending on model specification) suggests that, after controlling for school- and individual-level confounders, students in this group outperform the reference group by an amount roughly equivalent to one-quarter to one-half of the observed father-education gap. These differences, though not dominant in explaining overall variance, are sufficiently large to be educationally observable and actionable for school-based support strategies.

### 3.3. Analysis of Reasons for Poorer Academic Performance of Urban Primary School Students with Highly Educated Mothers and Non-Highly Educated Fathers

Previous analysis found that mother-highly educated/father-not students perform worse than both-parents-non-highly educated students. Research grounded in family investment and stress models shows parental involvement mediates relationships between parental education and children’s performance (Z. Li, 2013; Sohr-Preston et al., 2013). Jhang and Lee (2018) found home-based involvement and expectations significantly influenced both initial achievement and growth rates, with manifestations varying across contexts (Bartoli et al., 2022). Parental education is associated with educational participation (Fantuzzo et al., 2000; X. Li & Zheng, 2017). Highly educated parents, having already enjoyed the conveniences and benefits brought by advanced human capital or cultural capital, hope their children can also enjoy these advantages, thus having higher educational expectations for their children (Qiu & Xiao, 2011) and investing more educational participation and behavioral support, such as correcting children’s learning attitudes and cultivating good learning habits to promote children’s academic performance (Zhao & Hong, 2013). However, consistent with family stress model predictions (Conger et al., 2010), excessive intervention through strict discipline can harm performance (M. Wang et al., 2017) and trigger rebellious psychology and anxiety (Sun et al., 1998). High expectations may cause excessive academic pressure and test anxiety, harming performance (Levpuscek & Zupancic, 2009; Y. Wang et al., 2018).

Research shows that compared to fathers, mothers care more about what happens to their children at school and their interpersonal relationships, undertake more educational participation work such as tutoring children’s homework, and the higher the mother’s education, the higher the level of educational participation (Liu, 2019). Based on this, we speculate that compared to urban primary school students whose parents are both non-highly educated, mothers of urban primary school students whose mother is highly educated and whose father is non-highly educated may have higher educational expectations for their children and may have excessive intervention behaviors in their children’s learning, which may lead to excessive academic pressure on children. Additionally, fathers in families with highly educated mothers and non-highly educated fathers typically participate less in their children’s learning affairs, and fathers’ learning participation level has a positive promoting effect on children’s academic performance (Nord, 1998), which will therefore be detrimental to children’s academic performance. Based on this, we will examine whether fathers’ lower educational participation level and children’s greater academic pressure or more severe test anxiety are reasons for the poorer academic performance of urban primary school students whose mother is highly educated and whose father is non-highly educated.

Table 4 shows that through *t*-tests, there is no significant difference in overall parental learning participation level between families with highly educated mothers and non-highly educated fathers and families where both parents are non-highly educated (*p* > 0.1). However, compared to families where both parents are non-highly educated, fathers’ learning participation level is significantly lower in families with highly educated mothers and non-highly educated fathers (*p* < 0.05). Regarding mothers’ learning participation, compared to families where both parents are non-highly educated, mothers’ learning participation level is significantly higher in families with highly educated mothers and non-highly educated fathers (*p* < 0.01). Additionally, compared to families where both parents are non-highly educated, urban primary school students with highly educated mothers and non-highly educated fathers experience greater academic pressure, but there is no significant difference between the two (*p* > 0.1).

**Figure 3:**
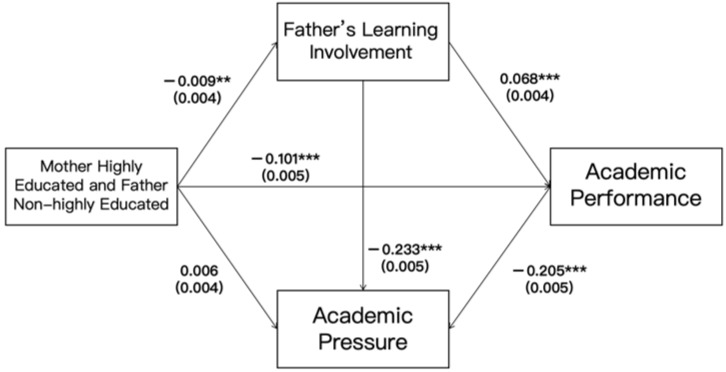
Path analysis of reasons for poorer academic performance of children in families with highly educated mothers and non-highly educated fathers. Note: **, *** indicate coefficients significantly different from 0 at the 0.01 and 0.001 levels, respectively. Control variables (gender, only child status, boarding student status, and average family SES) are included but not shown. Models use maximum likelihood estimation with bootstrap standard errors.

We constructed path analysis models (Figure 3) to examine indirect pathways through which mother-hypergamy is statistically associated with children’s performance, focusing on fathers’ participation and children’s academic pressure as potential intermediaries (Jhang & Lee, 2018). Given the cross-sectional nature of our data, all path coefficients represent statistical associations rather than causal or developmental sequences. The chi-square values of the models are 109.996, with 8 degrees of freedom, and significance probability values *p* < 0.001. Although the fit standard was not reached, this is likely because the study’s large sample size makes the chi-square value easily affected. Referring to other indicators, CFI and TLI values are 0.990 and 0.976 respectively, and the RMSEA value is 0.015, indicating good model fit. Results show that compared to both parents being non-highly educated, having a highly educated mother and non-highly educated father is significantly and directly negatively associated children’s academic performance (β = −0.101, *p* < 0.001); having a highly educated mother and non-highly educated father is significant negative associated with fathers’ learning participation (β = −0.009, *p* < 0.05), while fathers’ learning participation is significantly positive associated with children’s academic performance (β = 0.068/0.060, *p* < 0.01), and the product of indirect path coefficients for “highly educated mother and non-highly educated father—fathers’ learning participation—academic performance” is −0.001, which is statistically significant at the 0.05 level.

Having a highly educated mother and non-highly educated father is positively related to children’s academic pressure (β = 0.006/0.001), but it is not statistically significant (*p* > 0.1), while academic pressure is significantly negatively related to children’s academic performance (β = −0.205/−0.197, *p* < 0.05), and the product of indirect path coefficients for “highly educated mother and non-highly educated father—academic pressure—academic performance” is −0.001, but it is not statistically significant (*p* > 0.1). Additionally, fathers’ learning participation is significantly negatively related to children’s academic pressure (β = −0.233, *p* < 0.001), and the product of indirect path coefficients for “highly educated mother and non-highly educated father—fathers’ learning participation—learning pressure—academic performance” is −0.0004, which is statistically significant at the 0.05 level.

## 4. Discussion

Based on large-scale academic monitoring data from a provincial monitoring project, this study primarily employs hierarchical linear models and path analysis to analyze the relationship between parental education levels and their matching patterns on urban primary school students’ academic performance, obtaining the following main research conclusions and proposing several recommendations accordingly.

### 4.1. The Detrimental Effect of Mother-Hypergamy Educational Matching on Children’s Achievement

Regarding parental education levels, higher overall parental or father’s education level correlates with better students’ academic performance; however, maternal education exhibits an “inverted U-shaped” relationship. For educational matching patterns, students with both parents highly educated or father highly educated and mother non-highly educated perform significantly better than both-parents-non-highly educated students, while mother-hypergamy students perform significantly worse.

These findings challenge linear assumptions about maternal education benefits and highlight the importance of considering family configurations (Pesando, 2022; Bai & Byun, 2016). The negative mother-hypergamy pattern is consistent with the notion that educational heterogamy deviating from traditional gender norms may coincide with family role tensions and inconsistent parenting practices that are associated with children’s outcomes (Blossfeld, 2009). When both parents are non-highly educated, they may be more willing to acknowledge shortcomings and allow autonomous learning. In highly educated mothers’ and non-highly educated fathers’ families, mothers’ maternal participation is observed to be significantly higher, and children’s academic pressure is also greater, suggesting a potential link between intensive maternal involvement and heightened student stress that warrants further investigation.

### 4.2. Co-Parenting Dynamics and the Role of Balanced Parental Involvement

Path analysis reveals that a highly educated mother and a non-highly educated father are significantly directly related to performance and significantly negatively related to fathers’ learning participation, while fathers’ learning participation is significantly positively associated with performance, with this indirect effect statistically significant. These findings support the family investment model’s emphasis on parental time and involvement importance (Yeung et al., 2002; Sohr-Preston et al., 2013), suggesting that educational heterogamy may be associated with suboptimal investment patterns.

These findings suggest that the observed negative association in mother-hypergamy families may be partly linked to an imbalance in co-parenting dynamics, particularly reduced paternal involvement, rather than reflecting a direct causal effect of maternal education per se. From the perspective of the family stress model, misaligned parental expectations and heightened academic pressure are associated with less favorable child outcomes (Conger et al., 2010; Finkelstein et al., 2007). This is further consistent with Kocsis et al. (2025), whose systematic review demonstrated that helicopter parenting which is often enacted by highly involved and well-intentioned parents, is negatively associated with students’ academic self-efficacy and positively associated with academic pressure and anxiety, underscoring that parental involvement, when excessive, may inadvertently undermine the very outcomes parents seek to promote.

These findings are also consistent with the theoretical lens of role conflict (Tian et al., 2025). In families where the mother is highly educated but the father is not, the traditional gender role expectation of ’men as breadwinners, women as homemakers’ is inverted. This deviation from traditional gender norms may threaten paternal identity, potentially leading to lower paternal involvement, while simultaneously placing an undue burden on the mother to manage the child’s education. However, we acknowledge that our study did not directly measure marital conflict, gender role disagreement, or co-parenting quality. Therefore, this interpretation remains theoretical and should be tested in future research with dedicated measures of family process dynamics.

### 4.3. The Crucial Role of Fathers’ Involvement in Reducing Children’s Academic Pressure

In highly educated mothers’ and non-highly educated fathers’ families, fathers’ learning participation is significantly lower, a pattern that is associated with poorer academic outcomes for children, while higher levels of paternal participation are related to better performance, potentially through alleviating children’s academic pressure. The findings invite reflection on traditional “male breadwinner, female homemaker” concepts. These results align with prior research demonstrating the importance of fathers’ involvement (Nord, 1998; Bartoli et al., 2022) and suggest educational heterogamy may be associated with traditional gender role patterns that are observed to be unfavorable for children’s outcomes.

With economic development, more women, like men, receive education and enter the labor market, making it increasingly appropriate for fathers to assume child-rearing responsibilities. For highly educated mothers’ and non-highly educated fathers’ families, spousal dynamics may differ from those in other family configurations. Potential tensions between strong maternal roles and traditional gender norms may coexist with marital conflicts in some cases. Rather than one parent being overly dominant or absent, a balanced division of labor and cooperation may be beneficial. Collaborative parenting, grounded in mutual understanding and support, may foster more favorable family environments.

A gender-balanced environment is often considered conducive to children’s personality formation and temperament development. In highly educated mother and non-highly educated father families, fathers may consider actively engaging in family education and increasing their learning-related participation to mitigate the potential pressure and anxiety associated with intensive maternal involvement. This possibility is supported by research demonstrating the complementary roles of parents in child development and the value of balanced involvement (Fantuzzo et al., 2000; Jhang & Lee, 2018). The importance of institutional facilitation of parental involvement is further underscored by Pusztai et al. (2025), who demonstrated that schools’ active efforts to build family-school-community partnerships significantly increase both home-based and school-based parental involvement. Their findings suggest that regular, mutual communication between schools and parents, along with community-building activities, can effectively reduce the disadvantage typically associated with lower parental education. This aligns with our policy recommendation that schools should adopt multifaceted, demand-sensitive strategies to engage parents from diverse educational backgrounds.

The lower paternal involvement observed in mother-hypergamy families may be viewed as a behavioral manifestation of gender norm deviation. As the family structure deviates from the traditional pattern, fathers may withdraw from an area (education) that is culturally perceived as the mother’s domain or where they feel less competent. Our findings imply that fostering a stronger co-parenting alliance, where both parents actively participate in educational activities regardless of their relative educational levels, could be a key strategy to mitigate the negative effects associated with such non-traditional educational matching patterns.

## 5. Limitations and Future Prospects

First, our path analysis is based on cross-sectional data. Due to data limitations, this study did not control for students’ pretest scores, making it impossible to isolate learning foundation impacts. Although relevant factors were controlled for, this study lacks experimental data, making it difficult to accurately reveal the causal relationship between parental education and its matching patterns and children’s academic performance. The indirect pathways we identified should be interpreted as statistical associations that are consistent with our theoretical model, not as evidence of causal mediation or developmental ordering. Longitudinal data are needed to establish temporal precedence and causal direction.

Second, while we have interpreted our findings through the lenses of cultural reproduction, family investment, role conflict, and gender norm theories, our observational data and analytical approach cannot definitively adjudicate between these competing theoretical explanations. For example, the lower performance of children in mother-hypergamy families could be due to the father’s withdrawal, the mother’s excessive pressure, or a combination of both. Future research, particularly employing experimental or quasi-experimental designs and richer longitudinal data on family processes such as marital quality, division of labor, and daily parenting stress, is needed to disentangle these mechanisms and provide a more comprehensive understanding (Conger et al., 2010; Schulz et al., 2017).

Third, although we have invoked role conflict and co-parenting theories to interpret the mother-hypergamy disadvantage, we did not directly measure key family process variables such as marital quality, division of parenting labor, gender role attitudes, or interparental conflict. The mediating pathways we tested were limited to paternal involvement and child academic pressure. Future studies should incorporate direct assessments of these family dynamics to more rigorously test the mechanisms underlying the observed association.

Finally, this study uses provincial monitoring data; although the sample is large, caution is needed when generalizing to other contexts. The core principle for interpreting our results is that conclusions should not be judged solely by *p*-values but comprehensively evaluated based on the consistency, replicability, and directional patterns of effect sizes. In this study, the robust disadvantage of the “father non-highly educated/mother highly educated” configuration across multiple model specifications and the moderate-to-large advantage of the “both highly educated” group provide empirically grounded guidance for identifying at-risk family configurations. While the pseudo-R^2^ values indicate that parental education matching is not the sole or dominant predictor of student achievement, the estimated score gaps are educationally meaningful. It is important to note that while the estimated differences are statistically robust due to the large sample size, the corresponding effect sizes only range from small to moderate. Therefore, these findings must never be overinterpreted as directly actionable prescriptions for school-based interventions. We recommend that policymakers and frontline practitioners treat our findings as complementary evidence and combine them with other family and school factors to design targeted interventions, rather than interpreting them as deterministic causal claims. The core value of these conclusions is to assist in identifying family configurations that may require additional support or focused attention, rather than directly providing intervention implementation plans. Two key research directions are worth promoting in the future: first, conducting cross-national and cross-cultural comparative studies to verify whether the patterns observed in this Chinese urban context can be extended to other educational and cultural settings (Blossfeld, 2009; Pesando, 2022); second, carrying out follow-up research with experimental or quasi-experimental designs to verify whether interventions targeting these family patterns can bring practically meaningful academic improvements.

## Figures and Tables

**Table 1:** Variable descriptions.

Variable	Variable Code	Variable Description
Student Academic Performance	SCO	Continuous variable. Composed by standardizing and averaging scores from three subjects: Chinese, Mathematics, and English.
Parental Education	PED	a. Father’s/Mother’s education. Categorical variable: junior high school, high school, undergraduate, graduate. In quantitative analysis, treated as a continuous variable. Junior high school and below = 9; High school = 12; Undergraduate = 16; Graduate and above = 19. In addition, we define parents’ high education as an undergraduate degree or above. b. Overall parental education level. Continuous variable. Composed by summing father’s education (continuous variable) and mother’s education (continuous variable). c. Parental educational matching pattern. Categorical variable: both parents highly educated; father highly educated and mother non-highly educated; mother highly educated and father non-highly educated; both parents non-highly educated. In quantitative analysis, using both parents’ non-highly educated as reference, treated as 3 dummy variables.
Gender	GEN	Binary variable. Male = 1; Female = 0
Only Child Status	SIN	Binary variable. Only child = 1; Non-only child = 0
Boarding Student Status	LSC	Binary variable. Boarding student = 1; Non-boarding student = 0
Students’ Average Family Socioeconomic Status	SESM	Continuous variable. Obtained by averaging individual student-level SES and aggregating to the school level. Student individual-level SES is composed by weighting standardized scores of family possessions, parental occupation, and parental education using the Analytic Hierarchy Process (AHP).
Teachers’ Average Teaching Experience	TEAM	Continuous variable. Obtained by averaging individual teacher-level teaching experience and aggregating to the school level.
School Support	SSL	Continuous variable. Composed by averaging six items including “The school ensures teachers have sufficient time to participate in professional development activities” and “The school provides sufficient professional growth opportunities at every stage of teacher development” (5-point scale from strongly disagree to strongly agree). The scale demonstrates good reliability and validity indicators (CR = 0.973, AVE = 0.859, CFI = 0.995, TLI = 0.991, RMSEA = 0.025).

**Table 2:** Hierarchical linear model estimation results of the impact of parental education on urban primary school students’ academic performance.

	Variable	(1)	(2)	(3)	(4)	(5)	(6)
Student Level	Father’s Education Level	2.658 ***	5.431 ***				
		(0.055)	(0.461)				
	Father’s Education Level ^2^		−0.102 ***				
			(0.017)				
	Mother’s Education Level			0.816 ***	4.905 ***		
				(0.054)	(0.464)		
	Mother’s Education Level ^2^				−0.151 ***		
					(0.017)		
	Overall Parental Education Level					1.057 ***	0.037
						(0.030)	(0.252)
	Overall Parental Education Level ^2^						0.019 ***
							(0.005)
	Gender	−5.602 ***	−5.552 ***	−5.828 ***	−5.762 ***	−5.638 ***	−5.668 ***
		(0.318)	(0.319)	(0.323)	(0.323)	(0.321)	(0.321)
	Only Child Status	11.461 ***	11.393 ***	12.465 ***	12.334 ***	11.746 ***	11.802 ***
		(0.336)	(0.336)	(0.340)	(0.340)	(0.339)	(0.339)
	Boarding Student Status	−20.036 ***	−20.025 ***	−20.711 ***	−20.705 ***	−20.424 ***	−20.378 ***
		(1.325)	(1.325)	(1.341)	(1.341)	(1.334)	(1.334)
School Level	Students’ Average SES	118.562 ***	119.146 ***	146.025 ***	146.306 ***	127.620 ***	127.133 ***
		(5.719)	(5.709)	(5.738)	(5.722)	(5.738)	(5.747)
	Teachers’ Average Teaching Experience	0.430	0.414	0.346	0.332	0.396	0.406
		(0.492)	(0.491)	(0.494)	(0.493)	(0.493)	(0.493)
	Teachers’ Average Teaching Experience ^2^	−0.002	−0.002	0.000	0.001	−0.001	−0.002
		(0.016)	(0.016)	(0.016)	(0.016)	(0.016)	(0.016)
	School Support	8.854 ***	8.852 ***	8.997 ***	8.990 ***	8.865 ***	8.865 ***
		(1.321)	(1.318)	(1.328)	(1.324)	(1.324)	(1.325)
	Constant	370.206 ***	352.237 ***	379.797 ***	353.704 ***	372.707 ***	386.026 ***
		(8.140)	(8.649)	(8.185)	(8.675)	(8.161)	(8.800)
	Student-level Variance	2034.561	2033.73	2087.304	2085.421	2062.204	2061.715
	School-level Variance	254.266	253.169	256.520	254.817	255.143	255.871
	ICC	0.111	0.110	0.109	0.109	0.110	0.110
	Student-level Pseudo R^2 a^	0.157	0.158	0.137	0.138	0.147	0.147
	School-level Pseudo R^2 a^	0.525	0.527	0.520	0.523	0.523	0.522
	Student-level Sample Size	81,460	81,460	81,460	81,460	81,460	81,460
	School-level Sample Size	985	985	985	985	985	985

Note: The calculation method for school-level and student-level pseudo R^2^ (effect size) refers to Snijders and Bosker (1994), as below. *** indicate coefficients significantly different from 0 at the 0.001 level. “Father’s/Mother’s/Overall Parental Education Level ^2^” means that the variable is the square of father’s/Mother’s/Overall Parental education level, as below.

**Table 3:** Estimation of the impact of different parental educational matching patterns on urban primary school students’ academic performance.

Variable	OLS Regression	Hierarchical Linear Model
Father Non-highly Educated/Mother Highly Educated	−11.486 *** (0.603)	−11.586 *** (0.529)
Both Parents Highly Educated	15.336 *** (0.400)	15.142 *** (0.388)
Father Highly Educated/Mother Non-highly Educated	11.857 *** (0.638)	11.689 *** (0.606)
Gender	−5.885 ***	−6.007 ***
	(0.326)	(0.311)
Only Child Status	12.647 ***	11.857 ***
	(0.337)	(0.328)
Boarding Student Status	−18.098 ***	−20.610 ***
	(1.195)	(1.289)
Students’ Average SES	124.581 ***	126.600 ***
	(1.846)	(5.743)
Teachers’ Average Teaching Experience	−0.088	0.465
	(0.170)	(0.493)
Teachers’ Average Teaching Experience ^2^	0.016 **	−0.003
	(0.006)	(0.016)
School Support	7.648 ***	9.229 ***
	(0.415)	(1.327)
Constant	406.549 ***	394.099 ***
	(2.591)	(8.173)
Adjusted R^2^	0.162	/
F	1733.95	
Student-level Variance	/	2048.780
School-level Variance		259.515
ICC		0.112
Student-level pseudo R^2^		0.161
School-level pseudo R^2^		0.529
Student-level Sample Size	86,088	86,088
School-level Sample Size	/	985

Note: **, *** indicate coefficients significantly different from 0 at the 0.01 and 0.001 levels, respectively. “Teachers’ Average Teaching Experience ^2^” means that the variable is the square of Teachers’ Average Teaching Experience, as below.

**Table 4:** Differences in related variables between families with highly educated mothers and non-highly educated fathers and families where both parents are non-highly educated.

		Mother Highly Educated and Father Non-Highly Educated	Both Parents Non-Highly Educated
Father’s Learning Involvement	Mean/SD	4.064/0.011	4.086/0.005
	Sample Size	10,717	40,333
	t	1.967 **	
Mother’s Learning Involvement	Mean/SD	4.478/0.008	4.435/0.004
	Sample Size	10,577	39,753
	t	−3.486 ***	
Parental Learning Involvement ^a^	Mean/SD	4.274/0.008	4.265/0.004
	Sample Size	10,335	38,880
	t	−0.423	
Academic Pressure ^b^	Mean/SD	1.727/0.012	1.707/0.006
	Sample Size	10,623	39,926
	t	−1.861	

Note: **, *** indicate coefficients significantly different from 0 at the 0.01 and 0.001 levels, respectively. ^a^ Father’s or mother’s learning involvement is composed of averaging three items: “Father/Mother often discusses school matters with me,” “Father/Mother often checks my homework,” and “Father/Mother pays attention to my test scores” (5-point scale). Parental learning involvement is composed of averaging father’s learning involvement and mother’s learning involvement. ^b^ Academic pressure is measured by the reverse-coded item “I can relax myself before exams” (5-point scale).

## Data Availability

Restrictions apply to the availability of these data. Data were obtained from the Jiangsu Basic Education Quality Monitoring Center and are available from the authors upon reasonable request with the permission of the Jiangsu Basic Education Quality Monitoring Center.

## Supplementary Materials

The following supporting information can be downloaded at: https://www.mdpi.com/article/10.3390/bs16071235/s1, Table S1: Graduate-Degree Definition: OLS and HLM Results; Table S2. Cohen’s d Effect Sizes for Academic Performance by Parental Education Pairing (Bachelor’s-Degree Definition); Table S3. Cohen’s d Effect Sizes for Academic Performance by Parental Education Pairing (Graduate-Degree Definition); Table S4. Variable Differences Between Mother-Graduate/Father-Non-Graduate and Both-Non-Graduate Families. Figure S1: Mean Academic Performance by Parental Graduate-Education Pairing; Figure S2: Mean Academic Performance by Father’s and Mother’s Graduate Status; Figure S3: Path Analysis of Lower Academic Performance in Mother-Graduate/Father-Non-Graduate Families.
