# Supplementary material for: Does Educational Assortative Mating Matter? Parental Education Matching and Children’s Academic Achievement in Urban China
Source: Behav Sci (Basel). 2026 Jul 20;16(7):1235. doi: 10.3390/bs16071235 (PMC13405836; doi:10.3390/bs16071235)
Supplement: Supplementary file 1 [file behavsci-16-01235-s001.zip › behavsci-4159429-supplementary.pdf]

Supplementary Materials

Table of Contents

Table S1 *Graduate-Degree Definition: OLS and HLM Results*……………………….S2

Table S2 *Cohen's d Effect Sizes for Academic Performance by Parental Education Pairing (Bachelor's-Degree Definition)*………………………………………..........S3

Table S3 *Cohen's d Effect Sizes for Academic Performance by Parental Education Pairing (Graduate-Degree Definition)*………………..……………..……….............S4

Table S4 *Variable Differences Between Mother-Graduate/Father-Non-Graduate and Both-Non-Graduate Families*……………………………………………...…..S5

Figure S1 *Mean Academic Performance by Parental Graduate-Education Pairing*……………………………………………………..……..………...……………S6

Figure S2 *Mean Academic Performance by Father's and Mother's Graduate Status*………………………………………………………..……..……………………..S7

Figure S3 *Path Analysis of Lower Academic Performance in Mother-Graduate/Father-Non-Graduate Families*……………………………………………………..……..…………………..S8

Notes for Appendix…………………………………………………..……..………..S9

Robustness Checks and Supplementary Analyses

Table S1: Graduate-Degree Definition — OLS and HLM Results

As a robustness check on the operationalisation of parental education, Table S3 replicates the main analysis using a graduate-degree threshold (master's degree or above; ≥ 19 years of schooling) to define "high education." Under this stricter definition, the group where the father holds a graduate degree and the mother does not shows a significant negative association with academic performance (OLS: −10.412, $p < 0.001$; HLM: −9.486, $p < 0.001$). Notably, the both-graduate group shows a positive and significant effect without SES control (OLS: 8.066, $p < 0.001$; HLM: 8.890, $p < 0.001$), while the father-non-graduate/mother-graduate group also shows a positive effect. The ICC is 0.111 and pseudo-R^2 is 0.141.

Table S1. Graduate-Degree Definition: OLS and HLM Results

	(1) OLS	(2) HLM
Father Non-highly Educated/Mother Highly Educated	−10.412*** (0.919)	−9.486*** (0.819)
Both Parents Highly Educated	8.066*** (0.611)	8.890*** (0.650)
Father Highly Educated/Mother Non-highly Educated	7.450*** (0.860)	8.522*** (0.850)
Gender	−6.055*** (0.330)	−6.177*** (0.315)
Only Child Status	13.790*** (0.341)	12.936*** (0.331)
Students' Average SES	−19.502*** (1.209)	−21.738*** (1.306)
Boarding Student Status	150.685*** (1.778)	151.588*** (5.748)
Teachers' Average Teaching Experience	−0.181 (0.172)	0.367 (0.496)
Teachers' Average Teaching Experience^2	0.019*** (0.006)	−0.001 (0.016)
School Support	7.713*** (0.421)	9.315*** (1.334)
Constant	397.255*** (2.624)	385.137*** (8.217)
N	86,088	86,088
R^2 / Pseudo R^2	0.139	0.141
School-Level Variance	—	261.819
Student-Level Variance	—	2107.239
ICC	—	0.111

Note. Standard errors in parentheses. * $p < 0.05$, ** $p < 0.01$, *** $p < 0.001$. Reference group: both parents non-graduate. Graduate education defined as master's degree or above (≥ 19 years). Pseudo R^2 calculated following Snijders & Bosker (1994).

Table S2: Effect Sizes (Cohen's *d*) — Bachelor's-Degree Definition

Table S4 reports Cohen's *d* effect sizes for pairwise comparisons of academic performance across parental education groups under the bachelor's-degree definition. Compared to the reference group (both parents non-highly educated), the *Both Parents Highly Educated* group shows the largest advantage ($d = -0.611$, large effect), followed by *Father Highly Educated/Mother Non-highly Educated* ($d = -0.359$, medium effect). The *Father Non-highly Educated/Mother Highly Educated* group exhibits a small negative effect ($d = 0.148$), indicating marginally lower performance than the reference group. The pairwise comparison between *Both Parents Highly Educated* group and *Father Non-highly Educated/Mother Highly Educated* group yields the largest effect size ($d = -0.774$), highlighting the substantial performance gap between the highest- and lowest-performing education pairing configurations.**Table S2. Cohen's *d* Effect Sizes for Academic Performance by Parental Education Pairing (Bachelor's-Degree Definition)**

Comparison	*n* (Group 0)	*n* (Group 1)	Cohen's *d*	95% CI
Father Highly Educated = 0 vs. 1	45,128	37,265	−0.595	[−0.609, −0.581]
Mother Highly Educated = 0 vs. 1	42,540	39,853	−0.359	[−0.373, −0.345]
Both Parents Highly Educated vs. Both Parents Non-Highly Educated	40,486	30,498	−0.611	[−0.627, −0.596]
Father Non-highly Educated/Mother Highly Educated vs. Both Parents Non-Highly Educated	40,486	9,355	0.148	[0.126, 0.171]
Father Highly Educated/Mother Non-highly Educated vs. Both Parents Non-Highly Educated	40,486	6,767	−0.359	[−0.385, −0.333]
Both Parents Highly Educated vs. Father Highly Educated/Mother Non-highly Educated	6,767	30,498	−0.249	[−0.276, −0.223]
Both Parents Highly Educated vs. Father Non-highly Educated/Mother Highly Educated	9,355	30,498	−0.774	[−0.798, −0.750]
Father Highly Educated/Mother Non-highly Educated vs. Father Non-highly Educated/Mother Highly Educated	9,355	6,767	−0.486	[−0.518, −0.454]

Note. Negative *d* values indicate that Group 1 scores higher than Group 0. Effect size benchmarks follow Cohen (1988): small ≈ 0.20, medium ≈ 0.50, large ≈ 0.80. High education defined as bachelor's degree or above (≥ 16 years).**Table S3: Effect Sizes (Cohen's *d*) — Graduate-Degree Definition**

Table S6 presents Cohen's *d* for the graduate-degree definition. The *Both Parents Highly Educated* group shows the largest advantage over the reference group (*d* = −0.515, medium-to-large effect), followed by *YFHML* (*d* = −0.418, medium effect). The *Father Highly Educated/Mother Non-highly Educated* group (*d* = 0.049) is essentially indistinguishable from the reference group in terms of raw mean performance, though regression results controlling for covariates reveal a significant

negative effect. Pairwise comparisons show that *Both Parents Highly Educated* outperforms *Father Non-highly Educated/Mother Highly Educated* substantially (d = −0.576).

Table S3. Cohen's *d* Effect Sizes for Academic Performance by Parental Education Pairing (Graduate-Degree Definition)

Comparison	n (Group 0)	n (Group 1)	Cohen's d	95% CI
Both Parents Highly Educated vs. Both Parents Non-Highly Educated	74,413	6,104	−0.515	[−0.541, −0.489]
Father Non-highly Educated/Mother Highly Educated vs. Both Parents Non-Highly Educated	74,413	3,381	0.049	[0.014, 0.083]
Father Highly Educated/Mother Non-highly Educated vs. Both Parents Non-Highly Educated	74,413	3,208	−0.418	[−0.453, −0.382]
Both Parents Highly Educated vs. Father Highly Educated/Mother Non-highly Educated	3,208	6,104	−0.104	[−0.147, −0.061]
Both Parents Highly Educated vs. Father Non-highly Educated/Mother Highly Educated	3,381	6,104	−0.576	[−0.619, −0.533]
Father Highly Educated/Mother Non-highly Educated vs. Father Non-highly Educated/Mother Highly Educated	3,381	3,208	−0.449	[−0.498, −0.400]

Note. Negative d values indicate that Group 1 scores higher than Group 0. Effect size benchmarks follow Cohen (1988): small ≈ 0.20, medium ≈ 0.50, large ≈ 0.80. Graduate education defined as master's degree or above (≥ 19 years).

Table S4: Differences in Related Variables Between Families with Highly Educated Mothers and Non-highly Educated Fathers and Families Where Both Parents Are Non-highly Educated (Graduate-Degree Definition)

Table S7 compares students whose families show the *Highly Educated Mothers and Non-highly Educated Fathers* pattern against the *Both Parents Are Non-highly Educated group* on five family involvement and stress indicators. Significant group differences emerge for father companionship (*FSCOMPANY*; $t = 2.850$, $p = 0.004$), mother companionship (*MSCOMPANY*; $t = −2.187$, $p = 0.029$), and tutoring frequency (*TUTORT*; $t = −13.736$, $p < 0.001$). Teacher companionship (*TSCOMPANY*; $t = 0.593$, $p = 0.553$) and reversed learning stress ($t = −0.650$, $p = 0.515$) show no

significant differences. Notably, the *YFLMH* group uses significantly more tutoring (*M* = 2.43 vs. 1.99), consistent with compensatory investment by graduate-educated mothers.

Table S4. Differences in Related Variables Between Families with Highly Educated Mothers and Non-highly Educated Fathers and Families Where Both Parents Are Non-highly Educated (Graduate-Degree Definition)

Variable	Both Parents Non-Highly Educated	Father Non-highly Educated/Mother Highly Educated	Mean Diff.	t	p
Father's Learning Involvement	4.124 (1.061), n = 86,593	4.075 (1.118), n = 4,044	0.049	2.850	0.004
Mother's Learning Involvement	4.480 (0.823), n = 85,414	4.509 (0.831), n = 4,007	−0.029	−2.187	0.029
Parental Learning Involvement	4.306 (0.811), n = 83,589	4.298 (0.825), n = 3,911	0.008	0.593	0.553
Academic Pressure	1.658 (1.180), n = 85,619	1.671 (1.215), n = 4,008	−0.012	−0.650	0.515

Note. Values reported as M (SD). Graduate education defined as master's degree or above (≥ 19 years). Equal-variance *t*-tests applied throughout.

Supplementary Figures

Figure S1 displays mean academic performance scores across the four parental education pairing groups under the graduate-degree definition. The "Both Graduate" group achieves the highest mean score, followed by the "Father Graduate / Mother Non-Graduate" and "Both Non-Graduate" groups. The "Father Non-Graduate / Mother Graduate" group scores the lowest, mirroring the bachelor's-degree pattern observed in the main analysis.

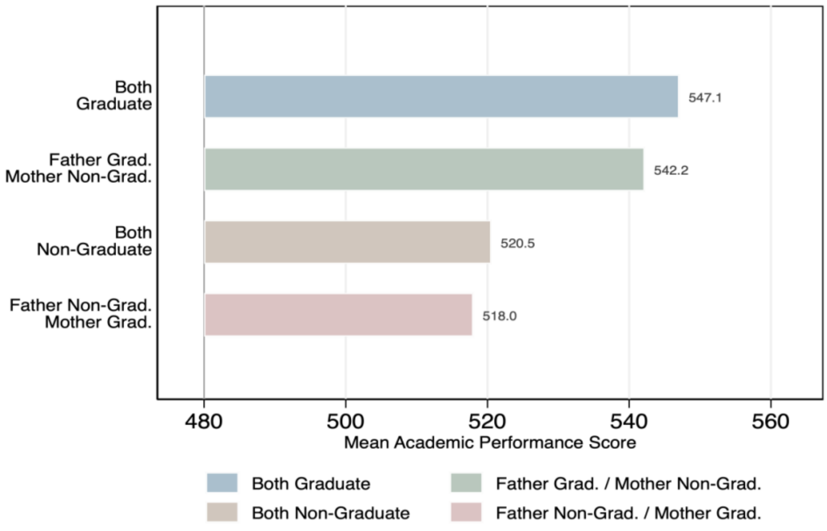

Figure S1 Mean Academic Performance by Parental Graduate-Education Pairing

Figure S2 presents mean academic performance scores separately for father's and mother's education under the graduate-degree binary definition. Father graduate status is associated with a substantially higher mean score than father non-graduate; the mother graduate vs. non-graduate gap follows a similar pattern but is comparatively smaller.

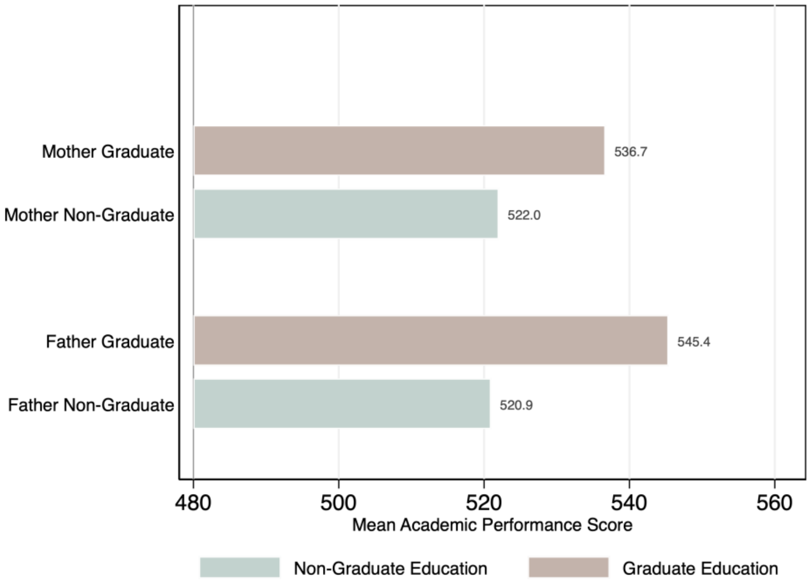

Figure S2 Mean Academic Performance by Father's and Mother's Graduate Status

Figure S3. Path Analysis of Reasons for Poorer Academic Performance of Children in Families with Highly Educated Mothers and Non-highly Educated Fathers **(Graduate-Degree Definition)**. Note: Control variables (gender, only child status, boarding student status, and average family SES) are included but not shown. Models use maximum likelihood estimation with bootstrap standard errors.

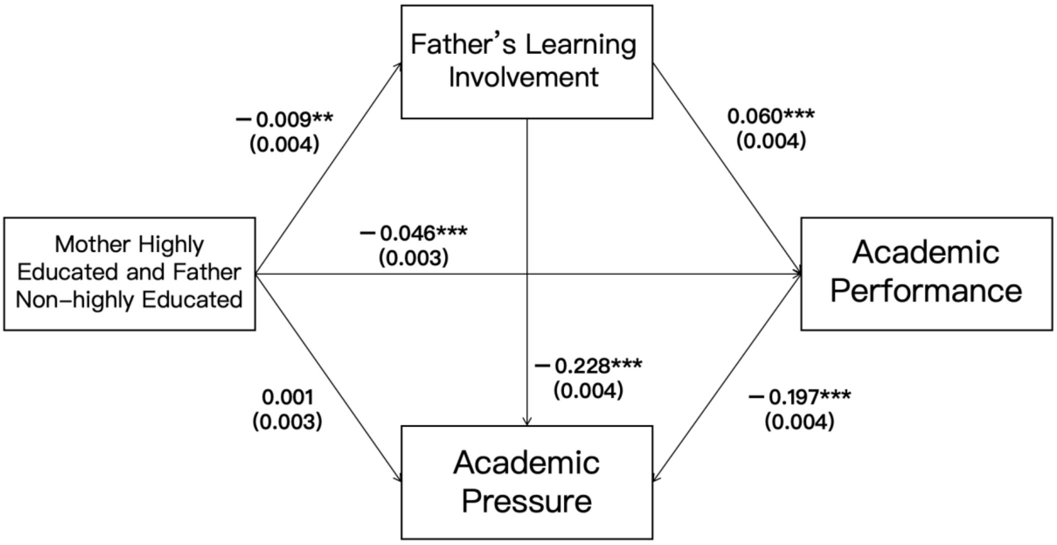

Figure S3 Path Analysis of Lower Academic Performance in Mother-Graduate/Father-Non-Graduate Families

Notes for Appendix. All models control for student gender, single-parent household status, boarding status, school-level SES, mean teacher experience and its square, and mean structural support at the school level, unless otherwise specified. Pseudo R^2 for hierarchical linear models is calculated following the method described in Snijders & Bosker (1994). Significance thresholds: $^{*}p < 0.05$, $^{**}p < 0.01$, $^{***}p < 0.001$.
